# Infliximab induced severe depression and suicidal thoughts in patient with bipolar disorder

**DOI:** 10.1192/j.eurpsy.2023.1607

**Published:** 2023-07-19

**Authors:** P. Ulual, H. S. Burhan, O. Güçlü

**Affiliations:** Psychiatry, Istanbul Basaksehir Cam ve Sakura Sehir Hastanesi, Istanbul, Türkiye

## Abstract

**Introduction:**

Infliximab is a tumor necrosis factor-alpha (TNF-α) inhibitor commonly used in the treatment of autoimmune disorders such as rheumatoid arthritis and ankylosing spondylitis. An increased risk of opportunistic infections, malignancy, and neurodegenerative diseases have been widely documented as adverse effects of IFX therapy. Few reports exist serving the notice of new-onset psychiatric symptoms linked to IFX treatment, such as suicidal behaviors in adults and elderly patients, as well as psychosis in an adolescent. Psychiatric side effects while under IFX treatment are reported to be rare.

**Objectives:**

Here, we present a case of a female with bipolar disorder who developed a long-standing depressive episode with suicidal thoughts after her fourth infusion of infliximab for her ankylosing spondylitis

**Methods:**

Retrospective life chart was created, including infliximab infusion.

Montgomery Asberg Depression Scale was applied at time of hospitalisation and discharge.

The Naranjo Adverse Drug Reaction Probability Scale was applied.

**Results:**

A 55 year old female with ankylosing spondylitis and bipolar disorder was treated with IFX for 8 months. During this period, a total of 4 infusions were administered and AS symptoms were well responding to the treatment. Patient describes the onset of depressive symptoms such as anhedonia and insomnia after the infusion of third IFX infusion, gradually progressing to loss of function and suicidal thoughts and hospitalization in a psychiatry clinic.

The patient had a history of bipolar disorder for 10 years with recurrent manic and depressive episodes, 4 hospitalisations and 1 cure of ECT.

Patient was on sertraline, maprotiline and diazepam at the time of hospitalization. We started treatment with aripiprazole, quetiapine and valproate, followed for 4 weeks as an inpatient, consulted with rheumatology treatment options and neurology for demyelinating disorders, no pathology was discovered. Rheumatology suggested the continuation of IFX infusion under psychiatric control. Fifth dose of IFX infusion was administered and patient was discharged after euthymic mood was established and insomnia and suicidal thoughts were deteriorated. Upon follow up, depressive symptoms recurred and lamotrigine was added for augmentation.

**Image:**

**Figure:**
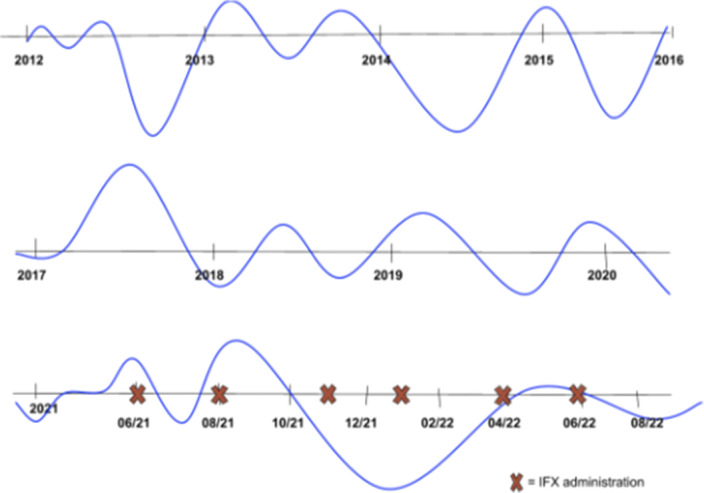

**Conclusions:**

Although there is very scarce evidence that IFX causes psychiatric symptoms, there are few clinical trials too, showing evidence that TNF-alpha inhibitors may improve depressive symptoms. While we need more information and evidence to support the ideas of TNF alpha inhibitors effects on human neuropsychology, it is of great importance for especially patients with psychiatric history to be closely watched while administering the product, at least to minimize unintended adverse events.

**Disclosure of Interest:**

None Declared

